# Physiological and Biochemical Dissection Reveals a Trade-Off between Antioxidant Capacity and Heat Tolerance in Bread Wheat (*Triticum aestivum* L.)

**DOI:** 10.3390/antiox10030351

**Published:** 2021-02-26

**Authors:** Mohammed Mohi-Ud-Din, Nurealam Siddiqui, Motiar Rohman, S. V. Krishna Jagadish, Jalal Uddin Ahmed, Mohamed M. Hassan, Akbar Hossain, Tofazzal Islam

**Affiliations:** 1 Department of Crop Botany, Bangabandhu Sheikh Mujibur Rahman Agricultural University, Gazipur 1706, Bangladesh; mmu074@bsmrau.edu.bd (M.M.-U.-D.); jahmed06@bsmrau.edu.bd (J.U.A.); 2 Department of Biochemistry and Molecular Biology, Bangabandhu Sheikh Mujibur Rahman Agricultural University, Gazipur 1706, Bangladesh; nuralambmb@bsmrau.edu.bd; 3 Plant Breeding Division, Bangladesh Agricultural Research Institute, Gazipur 1701, Bangladesh; mrahman@bari.gov.bd; 4 Department of Agronomy, Kansas State University, Manhattan, KS 66506, USA; kjagadish@ksu.edu; 5 Department of Biology, College of Science, Taif University, P.O. Box 11099, Taif 21944, Saudi Arabia; 6 Bangladesh Wheat and Maize Research Institute, Dinajpur 5200, Bangladesh; akbarhossainwrc@gmail.com; 7 Institute of Biotechnology and Genetic Engineering (IBGE), Bangabandhu Sheikh Mujibur Rahman Agricultural University, Gazipur 1706, Bangladesh

**Keywords:** heat stress, oxidative stress, reactive oxygen species, antioxidant, ascorbate-glutathione cycle, wheat

## Abstract

Heat stress alters photosynthetic components and the antioxidant scavenging system, negatively affecting plant growth and development. Plants overcome heat stress damage through an integrated network involving enzymatic and non-enzymatic antioxidants. This study aimed to assess physiological and biochemical responses in contrasting thermo-tolerant wheat varieties exposed to 25 °C (control) and 35 °C (heat stress), during the seedling stage. Our results revealed a substantial decrease in the photosynthetic pigments, carotenoids, anthocyanin content, and increased membrane injury index, malondialdehyde, methylglyoxal (MG), H_2_O_2_ contents and lipoxygenase activity compared to non-stress wheat seedlings. The heat-tolerant variety BARI Gom 26 (“BG26”) maintained higher cellular homeostasis compared to the heat susceptible variety Pavon 76 (“Pavon”), perpetuated by higher accumulation of proline, glycine betaine, ascorbate-glutathione cycle associated enzymes, reduced glutathione and ascorbate concentration in plant cells. Significantly lower levels of MG detoxification and antioxidant activities and ascorbate-glutathione cycle-related enzymatic activities lead to increased susceptibility in variety “Pavon”. Hierarchical clustering and principal component analysis revealed that variety “BG26” possess a combination of biochemical responses tailoring antioxidant activities that induced a higher level of tolerance. Taken together, our results provide a pipeline for establishing a trade-off between antioxidant capacity and heat tolerance to facilitate functional genomics and translational research to unravel underlying mechanisms to better adapt wheat to heat stress.

## 1. Introduction

Heat stress is one of the major environmental factors that can impact crop plants negatively, leading to impairment of several physiological and biochemical processes [1]. Global climate models predict that with increasing greenhouse gases, global mean surface temperatures are projected to increase by 0.3 to 4.8 °C by the end of the 21^st^ century [2]. Heat stress events lead to a significant yield loss in crops, including wheat under controlled environments [3,4] and also field conditions [5,6]. Heat stress is shown to have a significant negative impact during reproductive stages in crops, including wheat [7,8,9]. Comparatively, heat stress responses at the seedling stage in wheat are poorly understood, despite heat or combined heat and drought stress are known to adversely affect the early establishment of the wheat crop and seedling growth [10]. A large amount of wheat growing area in the north-western Bangladesh [11] and Indian Punjab [12] can be sown early, but early sown wheat crop suffers from juvenile heat stress which reduces tillers and seedling biomass, thereby negatively affecting grain yield [11].

To ascertain the degree of sensitivity or tolerance to heat stress in wheat, different screening techniques have been proposed. The difference in leaf tissue temperature compared to ambient temperature, also known as leaf temperature depression (LTD) has been recognized as a reliable indicator of heat tolerance [13]. A proportional increase in ion leakage with an increase in temperature has provided support to use ion leakage as an index for screening genotypes against heat and drought stress in wheat [14,15]. Almeselmani et al. [16] reported that an increase in membrane injury index (MII) in late-planted wheat genotypes helped capture the damaging effect of high temperature during the reproductive stage. The integrity and stability of plasma membrane measured in seedlings exposed to heat and drought stress were established as a reliable physiological marker for determining stress tolerance during later stages of crop development [17]. 

On exposure to heat stress, plants accumulate different signaling molecules or oxidative species and the degree to which plants can quench or balance their levels determines the degree of tolerance. The MG is a highly reactive mutagenic, cytotoxic and genotoxic αβ-dicarbonyl aldehyde compound, which is highly accumulated under extreme environmental stresses, including heat stress [18,19,20], which could act as a stress-responsive signal. MG is synthesized in biological systems via various enzyme-catalyzed and spontaneous reactions e.g., glycolysis, lipid and protein metabolism and can react with and modify both proteins and DNA, leading to the generation of advanced glycation end products (AGEs) [21,22,23]. At high cellular concentrations, MG inhibits cell proliferation and results in a number of adverse effects such as increasing the degradation of proteins and inactivating the antioxidant defense system [24,25].

The MG detoxification system is ubiquitously spread across cellular compartments to resist MG-mediated damage to the cellular constituents [23]. In plants, MG is detoxified mainly by the maintenance of GSH homeostasis via glyoxalase pathway containing enzymes, namely, glyoxalase I (Gly I) and glyoxalase II (Gly II) [26,27,28]. The Gly I converts MG to S-D-lactoylglutathione (SLG), utilizing GSH, while Gly II converts SLG to D-lactic acid and regenerates glutathione (GSH). Efficient detoxification of excess MG produced during normal physiological processes or different abiotic stresses is one of the most important adaptive strategies of plant stress tolerance [24,26,27].

In addition, heat stress accelerates the generation of reactive oxygen species (ROS) including singlet oxygen (^1^O_2_), superoxide anion (O_2_^•–^), hydrogen peroxide (H_2_O_2_) and hydroxyl radical (^•^OH), inducing oxidative stress in plants [29,30]. The main effects of ROS include autocatalytic peroxidation of membrane lipids and pigments, modification of membrane permeability and functions [31]. The level of lipid peroxidation has been widely used as an indicator of free radical damage to cell membranes under stress conditions. Malondialdehyde (MDA) is the principal and extensively studied product of polyunsaturated fatty acid (PUFAs) peroxidation. This aldehyde is a highly toxic molecule and needs to be considered more than just a marker of lipid peroxidation [32]. When PUFAs in bio-membranes are peroxidized, different aldehydes are formed including the highly reactive aldehyde MDA. The MDA is mostly generated in chloroplasts [33].

In general, responses of plants to heat stress may involve, among others, synthesis of various osmoprotectants, like proline and glycine betaine, heat shock proteins [34], and antioxidative enzymes to reduce oxidative damage [35]. To counteract the toxicity of ROS a highly efficient anti-oxidative defense system, composed of both non-enzymatic and enzymatic constituents, is required. The enzymatic antioxidant defense mechanisms are represented by enzymes that include, superoxide dismutase (SOD); four enzymes of the ascorbate-glutathione cycle: ascorbate peroxidase (APX), monodehydroascorbate reductase (MDHAR), dehydroascorbate reductase (DHAR) and glutathione reductase (GR); catalase (CAT); glutathione peroxidase (GPX); and glutathione s-transferase (GST) [36,37]. Non-enzymatic antioxidants include ascorbate (AsA), glutathione (GSH), tocopherol, flavanones, carotenoids, anthocyanins etc. [38,39]. Carotenoids and anthocyanins are known to be efficient quenchers of reactive oxygen species, such as peroxide radicals and singlet oxygen molecules and thereby alleviate the oxidative damage [40,41].

The generation, effects and activities of ROS, ROS scavengers and their interplay have been reported in the seedlings of different crop species including wheat exposed to heat stress. Most studies that have taken this route, have been mostly limited to single or two wheat genotypes [42]. The genotypes used in the above studies are either not suitable under current growing conditions or not included in current cropping patterns. Therefore, the overall aim of this study is to achieve results that are relevant to current cropping systems of Bangladesh and to address the gap in our understanding of heat stress responses during the seedling stage of wheat. To achieve this, we have selected four differentially heat-tolerant (ability of a cultivar to sustain yield under heat stress conditions) wheat varieties and quantified the physiological and biochemical attributes to gain a mechanistic insight associated with heat-induced oxidative stress tolerance. 

Specific objectives of this study were formulated to: (i) determine changes in morphological and physiological traits and pigment content in the selected wheat varieties exposed to heat stress; (ii) measure variation in the accumulation of oxidative stress indicators in response to heat stress; (iii) determine changes in oxidative stress alleviating antioxidants in wheat varieties under heat stress; and (iv) determine if an association exists between antioxidant activities and heat tolerance by comparing the responses in wheat varieties contrasting for heat stress responses.

## 2. Materials and Methods

### 2.1. Plant Materials and Stress Treatment

Four wheat varieties differing in heat stress tolerance viz., a high yielding, widely cultivated and heat tolerant BARI Gom 26 [“BG26”] [43,44], moderately heat and salinity tolerant BARI Gom 25 [‘BG25’], moderately heat-tolerant BARI Gom 23 (Bijoy) [‘BG23’] [43] and a widely used highly heat susceptible variety Pavon 76 [“Pavon”] [44] were used (Table S1).

Uniform sized seeds of four wheat varieties were selected and surface-sterilized with 70% ethanol followed by washing several times with sterile distilled water. The seeds were then soaked in distilled water for 10 min and sown in Petri plates (15 cm diameter) filled with sterile sand moistened with distilled water for germination for 3 days in the laboratory at a variable temperature of 26 to 32 °C. After germination, seedlings were then moved to controlled environment chamber maintained at 25 ± 1 °C during day and night, relative humidity (RH) of 75–80%, 16 h of photoperiod with a light intensity of 200 μmol photon m^−2^ s^−1^, about 1/10th full light intensity. After 5 days, two sets of seedlings (5 Petri plates for each variety with 20 seedlings in each plate) were then grown in two growth chambers with constant temperatures of 25 ± 1 °C (control) and 35 ± 1 °C (heat stress) with RH 75–80%, 16 h photoperiod with a light intensity of 200 μmol photon m^−2^ s^−1^ for 48 h. Growth chamber temperature and RH were monitored by a digital humidity and temperature meter (Model: HD-306, HTC Instruments, Taipei, Taiwan). Petri plates were irrigated every day with half-strength Hoagland’s nutrient solution. 

After termination of heat stress, shoot and root length and dry matter was recorded. Shoot and root length was measured from the root-shoot junction to the tip of the longest leaf and root, respectively. Dry matter was measured after oven drying the shoot and root at 60 °C for at least 2 days, till a constant weight was achieved. Before harvesting fully expanded leaves were collected into liquid nitrogen and used for pigment determination and biochemical assays. Expanded leaves from about 20 seedlings were collected to form a single replicate and the same repeated twice from an independent set of seedlings to obtain three biological replicates. All the measurements and assays were done in triplicate. Average of three biological replicates are presented in Tables and Figures.

### 2.2. Determination of Chlorophyll, Carotenoid and Anthocyanin Content 

Chlorophyll (Chl) content was determined by taking fresh fully opened leaf samples (0.2 g) from randomly selected seedlings. The samples were homogenized with 5 mL of acetone (80% *v/v*) using pre-cooled pestle and mortar and the homogenate was centrifuged at 5000× *g* for 10 min. The absorbance was measured with a UV-visible spectrophotometer (Genesys 10S UV-VIS, Thermo Fisher Scientific, Waltham, MA, USA) at 663 and 645 nm and Chl contents were calculated using the equations proposed by Arnon [45]. 

Carotenoids content was estimated according to the procedure described by Lachman et al. [46] and Rahman et al. [47] with slight modifications. Briefly, acetone extract was obtained by mixing 5 mL of HPLC grade acetone with 0.5 g homogenized leaf sample in a glass vial and allowed to stand for 24 h at 4 °C in the dark. The absorbance of the acetone extract was measured spectrophotometrically (Genesys 10S UV-VIS, Thermo Fisher Scientific, Waltham, MA, USA) against acetone at 444 nm. Total carotenoids content (mg g^−1^ FW of lutein equivalent) was calculated using the molar extinction coefficient of lutein.

Anthocyanin content was determined according to Hughes and Smith [48] and Rahman et al. [47] with some modifications. Briefly, 0.5 g of leaf sample was placed in the 5 mL solution of methanol, 6 M hydrochloric acid and distilled water (70:7:23) and extracted at 4 °C for 24 h in the airtight vials. To a 2 mL aliquot of the extract, 1 mL distilled water and 2 mL chloroform was added, the solution was vortex mixed and centrifuged at 5000× *g* for 15 min at 4 °C. The upper chloroform layer containing extracted anthocyanins was separated and the absorbance was measured with a UV-visible spectrophotometer (Genesys 10S UV-VIS, Thermo Fisher Scientific, Waltham, MA, USA) at 530 nm and the anthocyanin content (μg g^−1^ FW cyanidin-3-glucoside equivalent) was calculated using the molar extinction coefficient of cyanidin-3-glucoside.

The stability index (SI) of chlorophyll, carotenoid and anthocyanin was determined according to Sairam et al. [49] and calculated as follows- SI = (Pigment under stress/Pigment under control) × 100.

### 2.3. Leaf Temperature Depression (LTD)

Leaf temperature was measured by a hand-held infrared thermometer (Model- MT4, HTC Instruments, Taipei, Taiwan; distance-spot ratio 12:1) just before harvesting the seedlings for enzymatic assays. Measurements were taken maintaining an angle of approximately 30 °C to the horizontal line at a distance of 300 mm from the topmost fully opened leaf surface. Leaf temperature depression was calculated according to the method of Fischer et al. [50], as growth chamber temperature minus leaf temperature of the seedlings. Simultaneously, the temperature inside as the growth chamber was recorded from the digital humidity and temperature meter. Data for each replication was the mean of five readings.

### 2.4. Membrane Injury Index

Membrane injury index was determined by recording the electrical conductivity of leaf leachates in deionized water described by Deshmukh et al. [51]. Briefly, leaf samples (0.1 g) were cut into uniformly sized squares and placed in test tubes containing 10 mL of deionized water in two sets. One set was kept at 40 °C for 30 min and another set at 100 °C in boiling water bath for 15 min and their electric conductivities C_1_ and C_2_, respectively were measured by a conductivity meter (Model: EC-400L, Human Lab Instrument Co., Suwon, Korea).

(1) $$\mathrm{Membrane}\;\mathrm{injury}\;\mathrm{index}=\;\frac{C_{1}}{C_{2}}\times 100\;$$

### 2.5. Methylglyoxal

Samples were prepared according to Yadav et al. [26] with some modifications. One gram leaf tissue was homogenized in 5 mL of 0.5 M perchloric acid. After incubating for 15 min on ice, the mixture was centrifuged at 4 °C for 10 min at 11,000× *g*. The supernatant was decolorized by adding charcoal (10 mg mL^−1^), kept for 15 min at room temperature, and centrifuged at 11,000× *g* for 10 min. Before using this supernatant for MG assay, it was neutralized with a saturated solution of potassium carbonate keeping at room temperature for 15 min and centrifuged again at 11,000× *g* for 10 min. The neutralized supernatant was used for MG estimation.

MG was estimated according to the method described by Wild et al. [52]. Briefly, 20 µL of freshly prepared 500 mM ɴ-acetyl-ʟ-cysteine (in 100 mM sodium phosphate buffer, pH 7.0) was added to 980 µL of neutralized supernatant and incubated for 5 min at 22 °C. A control solution was prepared without adding neutralized supernatant. After incubation, absorbance at 288 nm was measured and control absorbance was subtracted. The final concentration of MG was calculated from the standard curve and expressed in terms of μmol g^−1^ FW.

### 2.6. Membrane Lipid Peroxidation and H_2_O_2_ Content

The level of membrane lipid peroxidation was measured by estimating malondialdehyde (MDA), a decomposed product of the peroxidized polyunsaturated fatty acid component of the membrane lipid, using thiobarbituric acid (TBA) as the reactive material following the method of Heath and Packer [53]. The leaf samples (0.5 g) were homogenized in 3 mL 5% (*w/v*) trichloroacetic acid (TCA) and the homogenate was centrifuged at 11,500× *g* for 10 min. One mL supernatant was mixed with 4 mL of TBA reagent (0.5% of TBA in 20% TCA). The reaction mixture was heated at 95 °C for 30 min in a water bath and then quickly cooled in an ice bath and centrifuged at 11,500× *g* for 15 min. The absorbance of the colored supernatant was measured at 532 nm and was corrected for non-specific absorbance at 600 nm. The concentration of MDA was calculated by using the extinction coefficient of 155 mM^−1^ cm^−1^ and expressed as nmol of MDA g^−1^ fresh weight.

H_2_O_2_ was assayed according to the method described by Yu et al. [54]. H_2_O_2_ was extracted by homogenizing 0.5 g of leaf samples with 3 mL of 50 mM K-phosphate buffer (pH 6.5) at 4 °C. The homogenate was centrifuged at 11,500× *g* for 15 min. Three mL of supernatant was mixed with 1 mL of 0.1% TiCl_4_ in 20% H_2_SO_4_ (*v/v*), and the mixture was then centrifuged at 11,500× *g* for 12 min at room temperature. The optical absorption of the supernatant was measured spectrophotometrically at 410 nm to determine the H_2_O_2_ content (є = 0.28 μM^−1^ cm^−1^) and expressed as μmol g^−1^ fresh weight.

### 2.7. Proline and Glycine Betaine

Free proline in leaf tissues was appraised spectrophotometrically using the acid-ninhydrin method following the protocol of Bates et al. [55]. The proline content was determined as μmol g^−1^ FW calculated from a standard curve. Glycine betaine (GB) content was measured spectrophotometrically by 1,2-dichloroethane method following the procedure of Valadez-Bustos et al. [56] and expressed as μmol g^−1^ FW calculated using a standard curve prepared from the series of known concentrations of betaine.

### 2.8. Ascorbate and Glutathione

Wheat leaves (0.5 g fresh weight) were homogenized in 3 mL of an ice-cold acidic extraction buffer containing 5% metaphosphoric acid and 1 mM EDTA using a mortar and pestle. Homogenates were centrifuged at 11,500× *g* for 15 min at 4 °C, and the supernatant was used for the analysis of ascorbate and glutathione following the methods described in Hasanuzzaman et al. [57].

### 2.9. Soluble Protein

Fresh leaf tissue (0.5 g) was homogenized in 1 mL extraction buffer containing 1 mM ascorbic acid, 1 M KCl, 0.5 M K-P buffer (pH 7.0), β-mercaptoethanol and glycerol in ice-cold mortar and pestle. The homogenate was centrifuged at 11,500× *g* for 15 min, and the supernatant was used as a soluble protein solution for enzyme activity. The protein concentration of each sample was determined following the method of Bradford [58] using BSA as a protein standard.

### 2.10. Assays for Antioxidant and Glyoxalase Enzyme Activity

The glyoxalase I (Gly I, EC: 4.4.1.5) assay was carried out according to Hasanuzzaman et al. [57]. Briefly, the assay mixture contained 100 mM K-phosphate buffer (pH 7.0), 15 mM magnesium sulphate, 1.7 mM GSH and 3.5 mM MG in a final volume of 700 μL. The reaction was started by the addition of MG and the increase in absorbance at 240 nm was recorded after 1 min of reaction. The activity was calculated using the extinction coefficient of 3.37 mM^−1^ cm^−1^.

The glyoxalase II (Gly II, EC: 3.1.2.6) activity was determined according to the method of Hasanuzzaman et al. [57] by monitoring the formation of GSH by measuring the change in absorption at 412 nm after 1 min. The reaction mixture contained 100 mM Tris-HCl buffer (pH 7.2), 0.2 mM DTNB and 1 mM S-D-lactoylglutathione (SLG) in a final volume of 1 mL. The reaction was started by the addition of SLG and the activity was calculated using extinction co-efficient of 13.6 mM^−1^ cm^−1^.

The superoxide dismutase (SOD, EC: 1.15.1.1) activity was assayed based on the competition between SOD and NBT for the production of superoxide from xanthine and xanthine oxidase interaction following Spitz and Oberley [59].

The lipoxygenase (LOX, EC: 1.13.11.12) activity was determined according to the method of Doderer et al. [60] using linoleic acid as a substrate solution. The increased absorbance was recorded at 234 nm after 1 min and the activity was calculated using an extinction coefficient of 25,000 M^−1^ cm^−1^.

The catalase (CAT, EC: 1.11.1.6) activity was measured according to the method of Hasanuzzaman et al. [57] by monitoring the decrease of absorbance at 240 nm after 1 min caused by the decomposition of H_2_O_2_. The reaction mixture contained 50 mM K-phosphate buffer (pH 7.0), 15 mM H_2_O_2_ and enzyme solution in a final volume of 700 μL. The reaction was initiated with enzyme extract and the activity was calculated using the extinction coefficient of 39.4 M^−1^ cm^−1^.

The guaiacol peroxidase (POD, EC: 1.11.1.7) activity was measured as described by Castillo et al. [61]. The reaction mixture contained 10 mM phosphate buffer at pH 6.1, 12 mM hydrogen peroxide, 96 mM guaiacol and enzyme extract. The blank contained a complete reaction mixture without H_2_O_2_. Absorbance was recorded at 470 nm after 1 min and the activity was calculated using the extinction coefficient of 26.6 mM^−1^ cm^−1^.

The glutathione peroxidase (GPX, EC: 1.11.1.9) activity was measured as described by Elia et al. [62] using H_2_O_2_ as a substrate. The reaction mixture consisted of 100 mM Na-phosphate buffer (pH 7.5), 1 mM EDTA, 1 mM NaN_3_, 0.12 mM NADPH, 2 mM GSH, 1-unit GR, 0.6 mM H_2_O_2_ and sample solution. The reaction was started by the addition of H_2_O_2_. The oxidation of NADPH was recorded at 340 nm for 1 min and the activity was calculated using the extinction coefficient of 6.62 mM^−1^ cm^−1^.

The glutathione S-transferase (GST, EC: 2.5.1.18) activity was determined spectrophotometrically by the method of Hasanuzzaman et al. [57] with some modifications. The reaction mixture contained 100 mM Tris–HCl buffer (pH 6.5), 1.5 mM GSH (reduced glutathione), 1 mM 1-chloro-2,4-dinitrobenzene (CDNB), and enzyme solution in a final volume of 700 μL. The enzyme reaction was initiated by the addition of CDNB and the increase in absorbance was measured at 340 nm after 1 min. The activity was calculated using the extinction coefficient of 9.6 mM^−1^ cm^−1^.

The glutathione reductase (GR, EC: 1.6.4.2) activity was measured according to Hasanuzzaman et al. [57]. The reaction mixture contained 0.1 M K-P buffer (pH 7.0), 1 mM EDTA, 1 mM GSSG, 0.2 mM NADPH, and enzyme solution in a final volume of 1 mL. The reaction was initiated with GSSG and the decrease in absorbance at 340 nm was recorded after 1 min. The activity was calculated using an extinction coefficient of 6.2 mM^−1^cm^−1^.

The ascorbate peroxidase (APX, EC: 1.11.1.11) activity was assayed following the method of Hasanuzzaman et al. [57]. The reaction buffer solution contained 50 mM K-phosphate buffer (pH 7.0), 0.5 mM AsA, 0.1 mM H_2_O_2_, 0.1 mM EDTA, and enzyme extract in a final volume of 0.7 mL. The reaction was initiated by the addition of H_2_O_2_ and activity was measured by observing the decrease in absorbance at 290 nm after 1 min using extinction co-efficient of 2.8 mM^−1^ cm^−1^.

The monodehydroascorbate reductase (MDHAR, EC: 1.6.5.4) activity was determined by the method of Hasanuzzaman et al. [57]. The reaction mixture contained 50 mM Tris-HCl buffer (pH 7.5), 0.2 mM NADPH, 2.5 mM AsA, 0.5 unit of AO and enzyme solution in a final volume of 700 μL. The reaction was started by the addition of AO. The activity was calculated from the change in absorbance at 340 nm after 1 min using an extinction coefficient of 6.2 mM^−1^cm^−1^.

The dehydroascorbate reductase (DHAR, EC: 1.8.5.1) activity was determined by the procedure of Hasanuzzaman et al. [57]. The reaction buffer contained 50 mM K-P buffer (pH 7.0), 2.5 mM GSH, and 0.1 mM DHA. The reaction was started by adding the sample solution to the reaction buffer solution. The activity was calculated from the change in absorbance at 265 nm after 1 min using an extinction coefficient of 14 mM^−1^cm^−1^.

### 2.11. Statistical Analysis 

All data obtained were subjected to 2-factor (treatment × varieties) analysis of variance (ANOVA) using the general linear model and the mean differences were compared using Tukey’s HSD test using the R packages *lme4* [63] and *agricolae* [64]. Differences at *p* < 0.05 were considered significant. The radar plot was prepared using R packages *fmsb* and *reshape2*. The stress tolerance index (STI) for all physiological and biochemical traits (Table S2) was calculated using the following formula: STI = [(X_c_ × X_s_)/(X̄_c_)^2^] [65], where X_c_ and X_s_ indicate the observed values of a trait in a given variety under non-stress and heat-stress treatments, respectively, while X̄_c_ is the average value of a particular trait examined in all varieties under non-stress condition. The library *pheatmap* of R version 4.0.2 was adapted to compute normalized mean values for generating heatmap and hierarchical clusters (distance = euclidean and method = ward.D2) [66]. The principal component analysis (PCA) was carried out using the packages *ggplot2*, *factoextra* and *FactoMineR* in R version 4.0.2 [67,68]. Correlation coefficient matrices were visualized using the R package *corrplot*. 

## 3. Results

### 3.1. Effect of Variety and Heat Stress on the Studied Traits

In the experimental setup including four wheat varieties and two growing conditions, all traits were significantly affected by both these factors but with different magnitudes (Table S3). Variation in LTD and Chl a/b were significantly affected by variety but not treatment. The interaction effect was significant in half the number of measured traits, but the contribution to the variation was less than the main effects.

### 3.2. Seedling Length and Dry Matter

**Figure 1:**
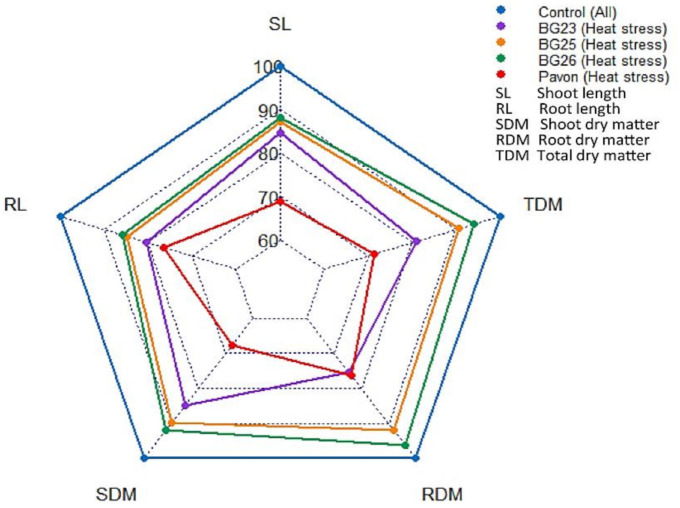
Radar plot showing changes in various morphological traits in different wheat varieties caused by heat stress. The values are expressed as % of the control.

Heat stress substantially reduced shoot length (SL) and root length (RL) of all varieties (Figure 1 and Figure S1). Compared with the control (100%), the reduction was the highest in Pavon (31% SL and 24% RL) and least in BG26 (12% SL and 15% RL), while in other two varieties the reduction was intermediate (Table S4). The dry matter of shoot (SDM), root (RDM) and the total dry matter (TDM) was reduced in all varieties due to heat stress (Figure 1). As a result of heat stress, the lowest TDM was recorded in “Pavon” (72% of the control) with the highest in “BG26” (94% of the control), while on average 86% TDM was recorded in other two varieties (Figure 1).

### 3.3. Chlorophyll, Carotenoid and Anthocyanin Content 

The Chl concentration in the wheat leaves decreased markedly under heat stress compared to control conditions in all varieties (Table 1). Compared to the control, Chl “a” content was decreased by 14, 16, 14 and 26% in “BG23”, “BG25”, “BG26” and “Pavon”, respectively, due to heat stress. Similar to Chl ‘a’, total Chl also decreased upon exposure to heat stress where the reduction was lower in the heat-tolerant “BG26” (13%) compared to heat susceptible “Pavon” (21%). Chl “b” and Chl a/b, however, remained statistically unchanged due to heat stress in all varieties. Carotenoid content in the wheat leaves decreased markedly due to heat stress in all varieties though the decrease was not statistically significant (Table 1). Anthocyanin content in the wheat leaves decreased significantly due to heat stress in all varieties (Table 1). The pigment was decreased by 22, 16, 15 and 29% in “BG23”, “BG25”, “BG26” and “Pavon”, respectively, upon exposure to heat stress. The stability of the pigments was higher in heat-tolerant “BG26” and lower in “Pavon” (Table 1). 

### 3.4. Leaf Temperature Depression and Membrane Injury Index

**Figure 2:**
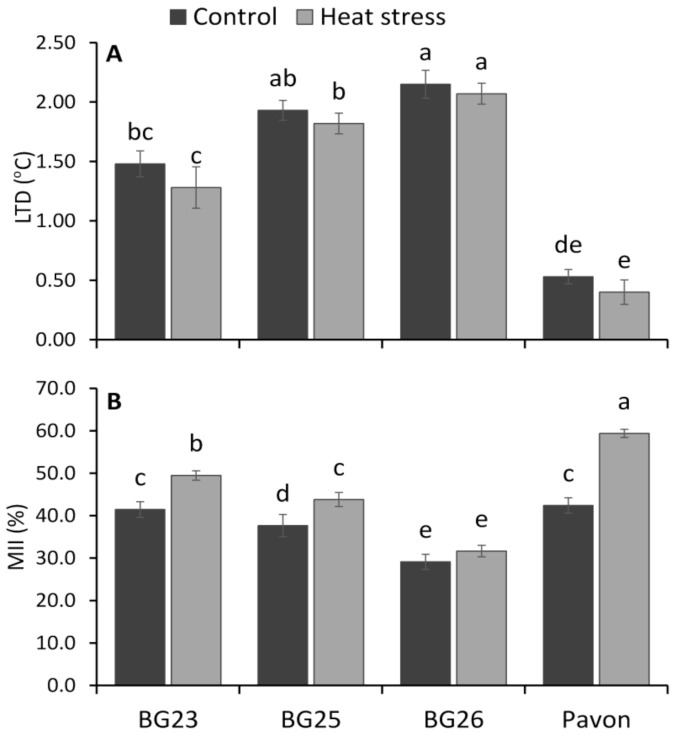
(**A**) Leaf temperature depression (LTD) and (**B**) membrane injury index (MII) of wheat varieties grown in the growth chamber under control and heat-stressed conditions. Vertical bars represent +/− SE values. Different letter(s) indicate significant difference at *p* < 0.05.

In this study, leaf temperature depression (LTD) in all wheat varieties was decreased under heat stress (35 °C) conditions compared to control (25 °C) (Figure 2A). Among the tested varieties, “BG26” maintained relatively higher LTD (2.15 and 2.07 °C in control and heat stress, respectively) while “Pavon” showed comparatively lower LTD (0.53 and 0.40 °C) under both growing conditions. The LTD were decreased by 14, 6, 4, and 25% in “BG23”, “BG25”, “BG26” and “Pavon”, respectively, under heat stress compared to their respective control seedlings, but none of them differed significantly between treatments. There was a significant increase in membrane injury index (MII) in all wheat varieties except “BG26” under heat stress conditions. “Pavon” recorded a maximum increase in MII on exposure to heat stress compared to other varieties whereas, “BG26” had the lowest increase in MII under heat stress (Figure 2B). The percentage increase in MII under heat stress compared to control was 19, 16, 9 and 40% in “BG23”, “BG25”, “BG26”, and “Pavon”, respectively.

### 3.5. Membrane Lipid Peroxidation, LOX Activity and H_2_O_2_ Level

**Figure 3:**
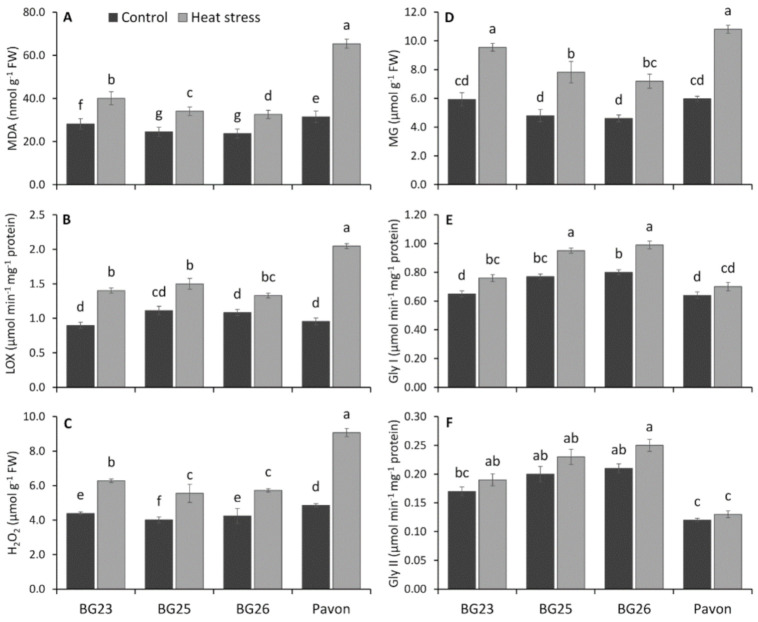
Changes in- (**A**) malondialdehyde (MDA), (**B**) lipoxygenase (LOX), (**C**) hydrogen peroxide (H_2_O_2_), (**D**) methylglyoxal (MG) content and the specific activity of (**E**) glyoxalase I (Gly I) and (**F**) glyoxalase I (Gly II) enzymes of wheat varieties grown under control and heat-stressed conditions. Vertical bars represent +/− SE values. Different letter(s) indicate significant difference at *p* < 0.05.

The membrane lipid peroxidation levels in leaf tissues measured as the MDA content increased significantly in seedlings exposed to heat stress, irrespective of the wheat variety (Figure 3A). Under heat stress conditions, the relative increase in MDA content was 42, 39, 38 and 108% in “BG23”, “BG25”, “BG26” and “Pavon”, respectively, compared to control. A greater increase (108%) of MDA content was recorded in variety “Pavon” compared to control, indicated higher leakiness, lower heat-stability and higher fluidity of membrane compared to other varieties. On average, the other three varieties recorded 39.5% increase in MDA.

The LOX activity of wheat leaves recorded a significant increase in all tested varieties, upon exposure to heat stress (Figure 3B). As a result of heat stress, the least (23%) increase in LOX activity was recorded in “BG26” with the highest (114%) in “Pavon”, while on average 46% increase was recorded in other two varieties. 

A significant increase in the cellular H_2_O_2_ level was observed in wheat leaves in response to heat stress compared to control (Figure 3C). Upon heat treatment, the amount of H_2_O_2_ increased by 43, 39, 35, and 87% in “BG23”, “BG25”, “BG26” and “Pavon”, respectively, compared to control. The lower relative increase in H_2_O_2_ level in ‘BG25′ and “BG26” indicates lower cellular toxicity and oxidative damage compared to ‘BG23′ and “Pavon”.

### 3.6. Methylglyoxal Level and Detoxifying Enzymes

The MG levels varied slightly and ranged from 4.60 to 5.97 μmol g-1 FW under control, but the level increased significantly due to heat stress and the levels ranged from 7.19 to 10.81 μmol g-1 FW between the wheat varieties (Figure 3D). The relative increase in MG level were 61, 63, 56 and 81% in “BG23”, “BG25”, “BG26”, and “Pavon”, respectively relative to control. The higher relative increase in MG level in “Pavon” indicated higher cellular toxicity, increased degradation of membrane proteins, lipids and nucleic acid under heat stress. 

Increase in Gly I activity was observed in all wheat varieties in response to heat stress, with a significant increase in “BG23”, “BG25” and “BG26”, but not in “Pavon” (Figure 3E). Heat stress resulted in 17, 22, 25, and 10% increase in Gly I activities in “BG23”, “BG25”, “BG26”, and “Pavon”, respectively, compared to control. 

The activity of Gly II was increased slightly with heat stress in all wheat varieties but the increase was non-significant (Figure 3F). As a result of heat stress, the highest increase (19%) in Gly II activity was recorded in “BG26” with the least (8%) in “Pavon”, while on average 14% increase was observed in other two varieties.

### 3.7. Activity of Osmolytes and Non-Enzymatic Antioxidants

**Figure 4:**
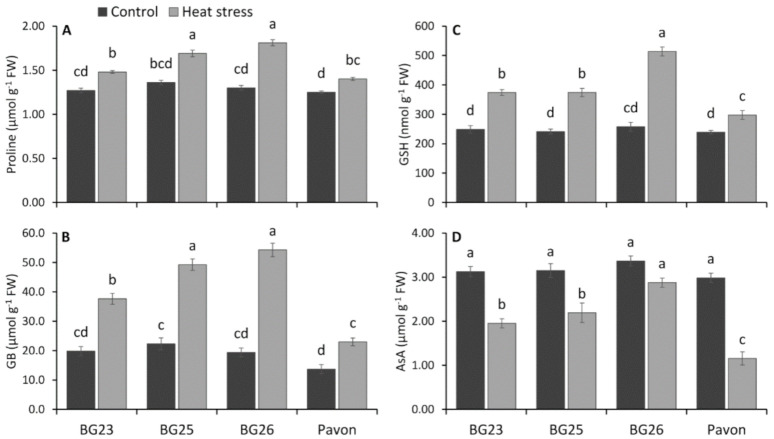
Changes in osmolytes and non-enzymatic antioxidants- (**A**) proline, (**B**) glycine betaine (GB), (**C**) glutathione (GSH) and (**D**) ascorbate (AsA) content of wheat varieties under control and heat-stressed conditions. Vertical bars represent +/− SE values. Different letter(s) indicate significant difference at *p* < 0.05.

Under control condition, statistically similar proline level was detected in all wheat varieties (Figure 4A). However, heat stress leads to a significant increase in proline accumulation in wheat seedlings of all four varieties. Upon exposure to heat stress, proline content increased by 17, 25, 39 and 12% in “BG23”, “BG25”, “BG26” and “Pavon”, respectively compared to control. 

Though statistically similar glycine betaine (GB) content, heat stress resulted in a substantial increase in GB content in all wheat varieties, compared to control (Figure 4B). GB content increased by 90, 121, 180 and 68% in “BG23”, “BG25”, “BG26” and “Pavon”, respectively, under heat stress conditions compared to control.

Similarly, glutathione (GSH) content was significantly increased under heat stress in all four tested wheat varieties (Figure 4C). On exposure to heat stress, “BG26” recorded 99% higher GSH content with the lowest increase (25%) recorded in “Pavon”, while other two varieties averaged at 54% increase in GSH. Leaf AsA content decreased significantly under heat stress in “BG25”, “BG23” and “Pavon”, but the reduction was not significant in “BG26” (Figure 4D). Under heat stress, “BG23” and “BG25” on average recorded 34% lower AsA, while “Pavon” recorded 61% lower AsA compared to 15% decrease in “BG26”.

### 3.8. Reactive Oxigen Species (ROS) Scavenging Enzymes

**Figure 5:**
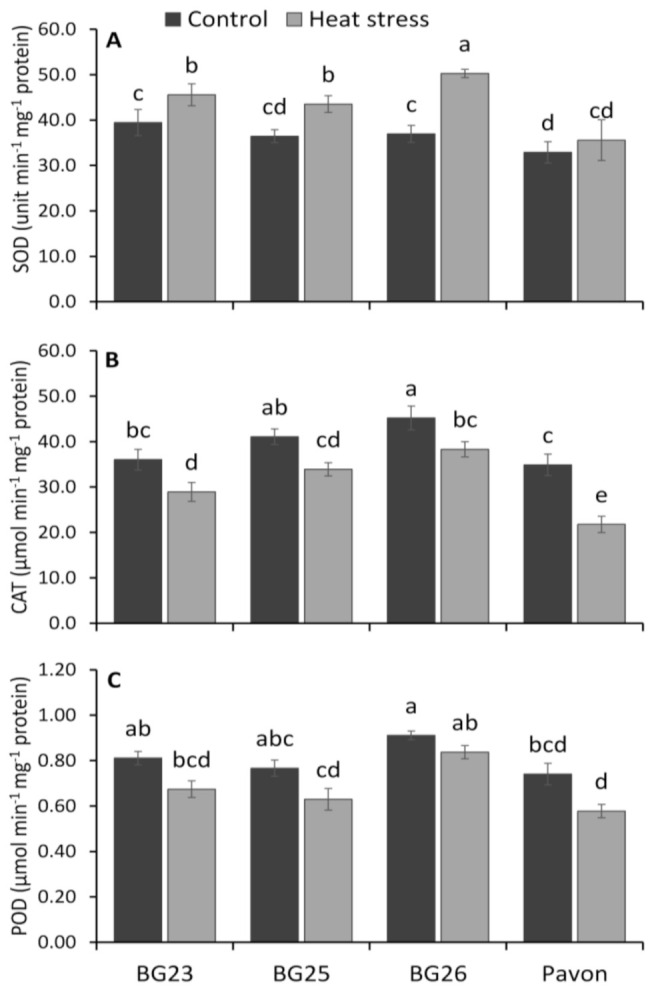
Specific activity of (**A**) superoxide dismutase (SOD), (**B**) catalase (CAT), and (**C**) guaiacol peroxidases (POD) enzymes of wheat varieties under control and heat-stressed conditions. Vertical bars represent +/− SE values. Different letter(s) indicate significant difference at *p* < 0.05.

In response to heat stress, an increase in SOD activity was observed in all wheat varieties with a significant increase in “BG23”, “BG25” and “BG26”, but not in “Pavon” (Figure 5A). Heat stress resulted in 16, 19, 36, and 8% increase in SOD activities in “BG23”, “BG25”, “BG26”, and “Pavon”, respectively, compared to control. 

The CAT activity was significantly decreased under heat stress in all the wheat varieties (Figure 5B), recording a decrease by 20, 18, 15, and 38% in “BG23”, “BG25”, “BG26”, and “Pavon”, respectively, compared to control. The lower relative decrease of CAT activity in “BG25”, “BG26” and “BG23” indicated an efficient and stable ROS scavenging system in these varieties than that of “Pavon”.

Similarly, POD activity was decreased non-significantly under heat stress in all wheat varieties (Figure 5C). Upon exposure to heat stress, the lowest (8%) decrease in POD activity was recorded in “BG26” and the highest (22%) in “Pavon” with an average of 17% in the other two varieties.

**Figure 6:**
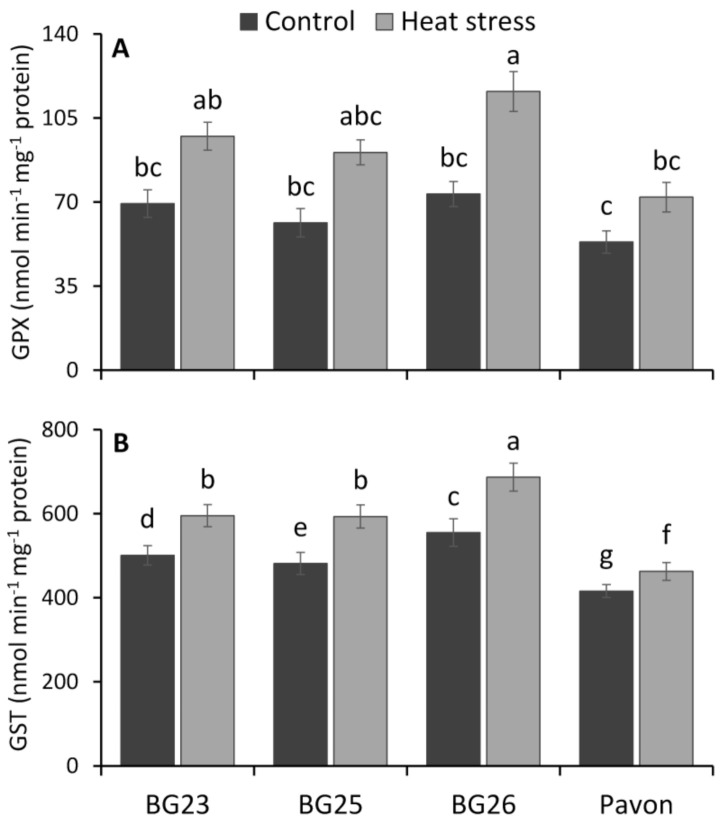
The specific activity of (**A**) glutathione peroxidase (GPX) and (**B**) glutathione-S-transferase (GST) enzymes of wheat varieties under control and heat-stressed conditions. Vertical bars represent +/− SE values. Different letter(s) indicate significant difference at *p* < 0.05.

Heat stress resulted in a significant increase in GPX activity in wheat seedlings of “BG26” but the increase was not significant in “BG23”, “BG25” and “Pavon” (Figure 6A). The highest GPX activity was recorded in “BG26” and the lowest in “Pavon” under both control and heat stress conditions. Due to heat stress, the GPX activity was increased by 41, 53, 61 and 38% in “BG23”, “BG25”, “BG26”, and “Pavon”, respectively, over control. GST activity of wheat leaves recorded a significant increase in all tested varieties, on exposure to heat stress (Figure 6B). As a result of heat stress, the lowest increase (11%) in GST activity was recorded in “Pavon” with the highest (24%) in “BG26”, while on average a 21% increase in other two varieties.

### 3.9. AsA-GSH Cycle Enzymes

**Figure 7:**
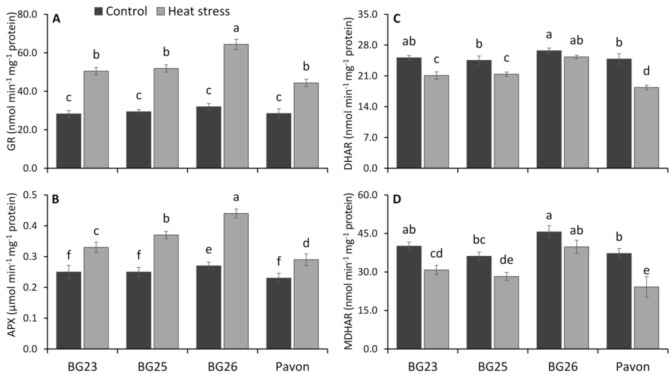
The specific activity of (**A**) glutathione reductase (GR), (**B**) ascorbate peroxidase (APX), (**C**) dehydroascorbate reductase (DHAR) and (**D**) monodehydroascorbate reductase (MDHAR) of wheat varieties under control and heat-stressed conditions. Vertical bars represent +/− SE values. Different letter(s) indicate significant difference at *p* < 0.05.

There was a significant increase in the glutathione reductase (GR) activity in all wheat varieties on exposure to heat stress (Figure 7A) and the increase ranged between 26 to 102% with “BG26” recording the highest increase and “Pavon” the least. Significant increase in APX activity was observed in all the tested varieties under heat stress (Figure 7B). Both under control and heat stress conditions, the highest APX activity was recorded in “BG26” and the lowest in “Pavon”. Under heat stress, the highest increase in APX activity was recorded in “BG26” (63%) with the least (26%) in “Pavon” while other two were intermediate with 32 and 48% increase in APX activity. 

Compared to the control, the activities of DHAR and MDHAR was decreased markedly in all wheat varieties due to heat stress (Figure 7C,D). Upon heat treatment, the activity of MDHAR decreased by 23, 22, 13 and 35% in “BG23”, “BG25”, “BG26” and “Pavon”, respectively, compared to control, without a significant decline only in “BG26”. The DHAR activity was also decreased in the same manner, and the decrease ranged between 5 to 26% with “BG26” having the lowest and “Pavon” recording the highest decline. 

### 3.10. Assessment of the Association between Treatment, Varieties and Variables Using Hierarchical Clustering and Principal Component Analysis (PCA) 

**Figure 8:**
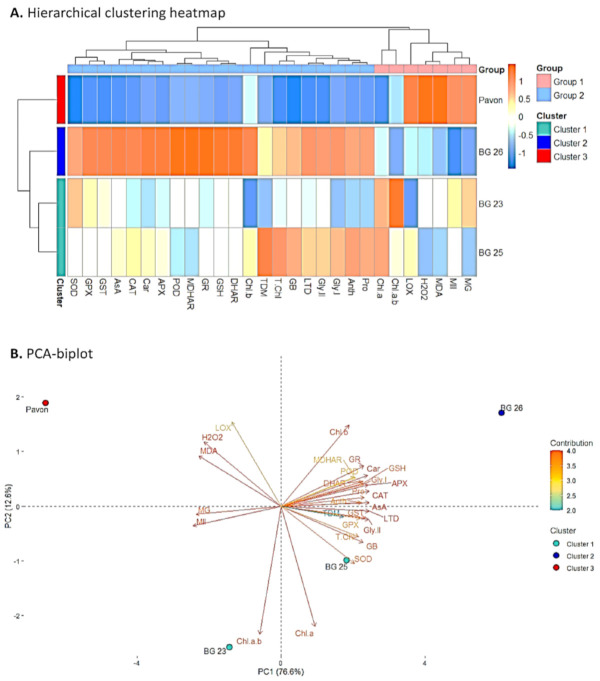
Hierarchical clustering and PCA-Biplot indicates an association between treatments, variables and varieties. (**A**) Hierarchical clustering heatmap: The STI (Stress Tolerance Index) mean values obtained from the studied variables of all varieties, which were then normalized and clustered. Two distinct groups were obtained at the variable levels (Group-1 and 2) (**B**) for all varieties. Different color scale expresses the intensity of the normalized mean values of various variables. PCA-Biplot: Varieties dispersed in different ordinates based on the dissimilarity among them. The length and color intensity of a vector in the biplot indicate the quality of representation and the contribution of the variable on the principal component, respectively. The angles between the vectors derived from the middle point of biplots exhibit positive or negative interactions among the variables. The variables included LTD, leaf temperature depression; MII, membrane injury index; MG, methylglyoxal; MDA, malondialdehyde; LOX, lipoxygenase; H_2_O_2_, hydrogen peroxide; Pro, proline; GB, glycine betaine; TDM, total dry matter; STI, stress tolerance index; GSH, glutathione; AsA, ascorbate; “Chl a”, chlorophyll *a;* “Chl b”, chlorophyll *b*; T.Chl, total chlorophyll; Chl.a.b, chlorophyll a/b; Car, carotenoids; Anth, anthocyanin; Gly.I, glyoxalase I; Gly.II, glyoxalase II; SOD, superoxide dismutase; CAT, catalase; POD, peroxidase; GPX, Glutathione peroxidase; GST, Glutathione *S*-transferases; GR, glutathione reductase; APX, ascorbate peroxidase; MDHAR, monodehydroascorbate reductase and DHAR, dehydroascorbate reductase.

The STI values of all measured physiological and biochemical variables were used to develop heatmap, hierarchical clustering and PCA. From the hierarchical clustering, two groups (Group-1 and -2) were obtained involving various physiological and biochemical variables measured among the wheat varieties (Figure 8A). Group-2 included all the measured variables, except MDA, H_2_O_2_, MII, LOX, MG, “Chl a” and Chl a/b which formed Group-1 (Figure 8A). In comparison to “BG26”, five of the variables in Group-1 exhibited a highly consistent increasing trend in “Pavon”, whereas Group-2 variables exhibited significantly decreasing pattern (Figure 8A). The other varieties like “BG23” and “BG25” showed a varying direction in changes among variables in both groups. Specifically, variety “BG26” displayed a decreasing trend in Group-1 and increasing trends in the variables of Group-2, which represented opposite trends in “Pavon” (Figure 8A). In addition, hierarchical clustering of the varieties revealed three distinct clusters namely Cluster-1, -2 and -3. “BG23” and “BG25” were placed in the Cluster-1 and were strongly associated with “BG26” compared to “Pavon” (Figure 8A). “BG26” and “Pavon” were positioned in the Cluster-2 and -3, respectively.

Subsequently, we performed PCA analysis using STI values to assess the association between varieties and variables (Figure 8B). The two PCA components PC1 and PC2 explained 78.8 and 12.7% of the total phenotypic variation, respectively (Figure 8B). Interestingly, the PCA results revealed that variables of Group-2 viz., DHAR, GR, GSH, SOD, Car, TDM, APX, AsA, CAT, LTD, Gly II, GB and T.Chl were the major contributors in PC1 and were strongly associated with “BG26”, while the variables from Group-1 which included H_2_O_2_, MDA, LOX, MG and MII were strongly associated with “Pavon” (Figure 8B). “Chl a” variable of Group-1 was found to be the most closely correlated with ‘BG25′, while ratio of “Chl a” to “b” was the most closely linked with “BG23” (Figure 8B).

## 4. Discussion

### 4.1. Pigment Stability Confers Judicious Light Absorption under Heat Stress 

Heat stress generates significantly higher levels of ROS including ^1^O_2_, O_2_^•−^, H_2_O_2_ and ^•^OH, thereby inducing oxidative stress in plants [29,69]. Excess energy that has not been used for photosynthesis will lead to higher amounts of ROS, which cause oxidative damage to chloroplasts and other cell structures when exposed to heat stress [70]. Heat stress also reduces Chl biosynthesis, the disintegration of chloroplast membranes and disruption of biochemical reactions in photosystems [71]. In this study, heat stress decrease in Chl content in wheat leaves could be attributed to impairment of Chl biosynthesis or rapid degradation [72,73,74]. Interestingly, the tolerant wheat variety “BG26” recorded greater stability of the photosynthetic pigments under heat stress compared to the heat susceptible “Pavon”, with a similar finding observed with late to very late-planted wheat [75]. Pigments stability particularly “Chl a” reflected by a stable Chl a/b ratio in the most tolerant “BG26” compared to other varieties, indicated its ability to sustain a higher proportion of the vulnerable “Chl a” content under heat stress (Table 1). 

Carotenoids protect photosystems and chlorophyll molecules by reacting with lipid peroxidation products and scavenging singlet oxygen [76]. Anthocyanins are known to protect cellular damage by stabilizing membrane fluidity [75,77,78] and act as effective antioxidant [79,80]. So, higher stability of these pigments under heat stress collectively indicates enhanced antioxidant system in tolerant wheat variety “BG26” compared to other varieties. This implies slower degradation of chlorophyll, carotenoids and anthocyanins, allowing stability in the light absorption machinery in “BG26” under heat stress (Table 1). Further, higher carotenoids and anthocyanins content would enhance heat tolerance in “BG26” through stable membrane system and effective ROS scavenging system.

### 4.2. Leaf Temperature Depression, Membrane Disruption and Cellular Toxicity Correlated with Oxidative Damage

High leaf temperature depression (LTD) or a cooler leaf has been used as a powerful and robust selection criterion to improve tolerance of plants to heat and drought stress [50,80,81,82]. LTD was the highest in “BG26”, closely followed by “BG25”, indicating the presence of an efficient transpiration cooling mechanism in these varieties compared to the other two (Figure 2A). LTD is an important heat avoidance mechanism [82] that helps to maintain higher assimilation rate in stress condition by improving stomatal conductance and protecting chloroplasts [80]. In addition to LTD, membrane injury index (MII) is a collective measure of cell membrane disruption due to stress and has been extensively used as a reliable marker for the estimation of stress-induced injury in plants [83]. Genotypes such as “BG26” (Figure 2B) having stable and functional cell membrane under heat stress are considered as promising candidates for further enhancing heat tolerance in wheat varieties [15,84].

**Figure 9:**
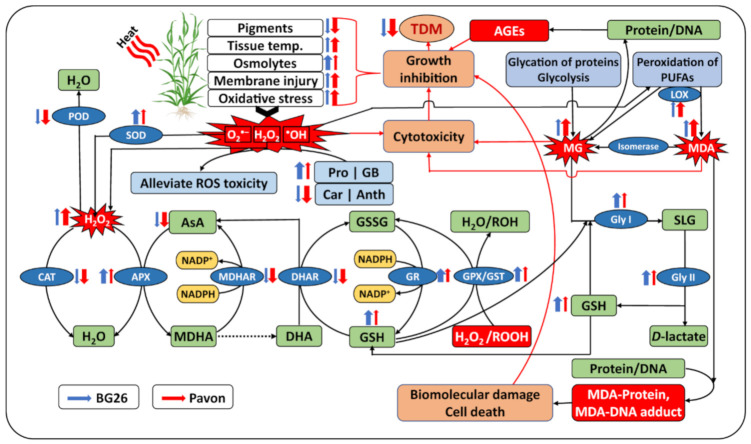
Schematic representation of the generation of ROS, MG and MDA due to heat stress and parametabolic reactions of AsA-GSH cycle and glyoxalase system in plants involved in ROS and MG detoxification. Red boxes and explosions indicate cellular oxidative stressors. Blue ellipses and green boxes represent the enzymes and substrate/product of the reactions, respectively. The blue and red arrows represent “BG26” and “Pavon”, respectively. The direction of the arrows indicate an overall increase (upwards) or decrease (downwards) on exposure to heat stress within a variety. Size of the arrow in terms of thickness indicates the amount/activity level with thicker arrows indicating the higher change (increase or decrease) under heat stress compared to control between the varieties. AGE- advanced glycation end product, Anth- anthocyanin, APX- ascorbate peroxidase, AsA- ascorbate, Car- carotenoids, CAT- catalase, DHA- dehydroascorbate, DHAR- dehydroascorbate reductase, DNA- deoxyribonucleic acid, GB- glycine betaine, Gly I- glyoxalase I, Gly II- glyoxalase II, GPX- Glutathione peroxidase, GR- glutathione reductase, GSH- glutathione (reduced), GSSG- glutathione (oxidized), GST- Glutathione *S*-transferases, H_2_O_2_- hydrogen peroxide, LOX- lipoxygenase, MDA- malondialdehyde, MDHA- monodehydroascorbate, MDHAR- monodehydroascorbate reductase, MG- methylglyoxal, NADPH- nicotinamide adenine dinucleotide phosphate (reduced), POD- peroxidase, Pro- proline, PUFA- polyunsaturated fatty acid, ROS- reactive oxygen species, SLG- *S*-D-lactoylglutathione, SOD- superoxide dismutase, and TDM- total dry matter.

**Figure 10:**
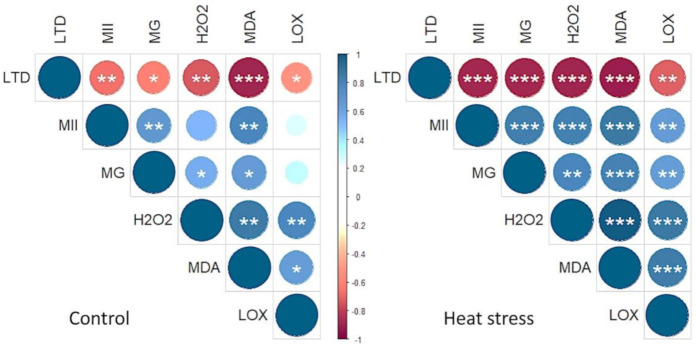
Correlation coefficients among the parameters that collectively induced oxidative stress. The parameters included LTD (leaf Table 2. O_2_ (hydrogen peroxide) and LOX (lipoxygenase). ***, ** and * indicate significant at *p* ≤ 0.001, *p* ≤ 0.01 and *p* ≤ 0.05, respectively.

H_2_O_2_ is a toxic compound which is harmful to cells, resulting in lipid peroxidation and membrane injury [85,86] and thereby produce highly reactive and cytotoxic aldehyde derivative MDA [87] (Figure 9). Elevated levels of MG are reported in different plants exposed to abiotic stressors [26]. The MDA levels and LOX activity are used as a measure of membrane lipid peroxidation and oxidative stress, and the impact it has on increasing membrane fluidity; increase in leakiness of the membrane; and damage to membrane proteins, enzymes, and ion channels [88,89]. Significant increase in the MG levels in “Pavon” indicates the extent of inactivation of the vital defense system and irreparable metabolic dysfunction under heat stress. In addition, a significantly higher H_2_O_2_, MDA and LOX levels in “Pavon” adds further support to a higher level of heat stress sensitivity, due to the inhibition or insufficient induction of the antioxidant defense systems compared to the tolerant “BG26”. MII, H_2_O_2_, MG, MDA and LOX collectively induce oxidative stress as these variables were significantly positively correlated under heat stress (Figure 10). Comparatively, a higher relative increase of Gly I and II in the heat-tolerant “BG26” (Figure 9) confers efficient MG detoxification in addition to playing a role in maintaining GSH homeostasis and subsequent ROS detoxification.

### 4.3. Enhanced Osmolytes Accumulation Alleviate Heat-Induced Oxidative Stress in Wheat 

Proline accumulation in plants has been associated with enhanced tolerance under different stresses, including unfavourable temperature conditions, either low or high temperatures [90,91]. As an important osmolyte, accumulation of proline is shown to alleviate osmotic stress induced by heat stress [92]. In this study, differences in the proline level between the wheat genotypes were not substantial but were markedly higher in plants exposed to heat stress at 35 °C compared to control (Figure 4A). This is consistent with other studies, where even higher increases have been reported in heat stress exposed wheat plants [93].

In vitro experiments have demonstrated glycine betaine (GB) in protecting some enzymes and protein complexes from heat-induced destabilization [94,95] and increased the levels of AsA and GSH in transgenic wheat by activating the synthesis of the molecules [95]. In the present study, higher accumulation of GSH (Figure 4C) and GB (Figure 4B) in the heat-tolerant “BG26” indicates lesser disruption of pathways synthesizing these antioxidants under heat stress compared to other varieties. Both glycine betaine and proline provide protection against oxidative stress by reducing H_2_O_2_ and lipid peroxidation levels and by increasing the antioxidant defense and MG detoxification systems [25].

### 4.4. Stable Antioxidant Enzyme System is Crucial for Heat Tolerance

In the plant cells, superoxide dismutase (SOD) provides primary protection against O_2_^•−^, which is then converted to H_2_O_2_ for subsequent metabolism to H_2_O by catalases (CAT) and peroxidases (POD, APX and GPX), thus protecting the cell damage [39,96]. In this study, the activity of both CAT and POD decreased significantly in all wheat varieties upon exposure to heat stress, which could be due to its inactivation by the accumulated H_2_O_2_ induced by heat. However, the extent of reduction varied with the relative tolerance of the varieties, i.e., comparatively higher reduction in susceptible “Pavon” than tolerant “BG26” (Figure 5A–C). The higher relative increase in SOD activity along with a lower relative decrease of CAT and POD activity may be beneficial to detoxifying O_2_^•−^ and H_2_O_2_ induced by heat stress in tolerant varieties, and vice versa in susceptible varieties. Our results align with other studies wherein a significant increase in SOD [97] and reduction in CAT [98,99,100,101] and POD activity under heat stress is observed, including wheat [102] and maize [103].

Glutathione peroxidases (GPX) are a family of isozymes that use GSH to reduce H_2_O_2_ and lipid hydroperoxides (ROOHs) (Figure 9), and therefore protect the plant cell membrane from oxidative damage [104,105,106]. Our results showed a sharp increase in GPX activity in response to heat stress with a relatively higher increase in the tolerant “BG26”, indicating the increased ability of the tolerant variety to scavenge H_2_O_2_ and ROOHs, similar to Hasanuzzaman et al. [107]. The plant glutathione-S-transferases (GST) are a large and diverse group of enzymes that catalyze the conjugation of electrophilic xenobiotic substrates with the GSH and are associated with inducing tolerance to different abiotic stresses [108]. More than 2-fold increase in GST activity was recorded in the tolerant “BG26” compared to the susceptible “Pavon” (Figure 6B), thereby decreasing the levels of MDA and H_2_O_2_ in wheat seedlings exposed to heat stress (Figure 3A,C). Similar increases in GST activity under heat stress were observed in wheat [18,107] and maize [109].

Taken together, apart from the AsA-GSH cycle, SOD, CAT, POD, GPX and GST are important antioxidant enzymes in plants. Our results demonstrated that the heat-tolerant “BG26” seedlings countered ROS production by maintaining a relatively higher amount of CAT and POD, and increasing the activities of SOD, GPX and GST under heat stress (Figure 5 and Figure 6).

### 4.5. Efficient Operation of Ascorbate-Glutathione Cycle is Pivotal for Heat Tolerance 

Ascorbate–glutathione cycle is the core metabolic pathway to detoxify ROS and recycling of non-enzymatic antioxidants. This vital cycle contains four enzymes: APX, MDHAR, DHAR and GR which are systematically and proportionately involved in the H_2_O_2_ detoxification (Figure 9). Other than H_2_O_2_ detoxification, these enzymes are also actively involved in the regeneration of non-enzymatic antioxidants like AsA and GSH. The presence of AsA and GSH has been reported to improve osmoregulation, plant water status and nutrient status, water use efficiency, photosynthetic performance, reduce oxidative stress and improve overall productivity in plants [4,109]. Our results demonstrate that AsA content markedly decreased under heat stress in all wheat varieties and it was less pronounced in heat-tolerant “BG26” (Figure 4D). A decline in AsA is probably due to its direct role in scavenging ROS, since AsA is considered to be the first line of defense against oxidative stress. The greater decline of AsA content in heat susceptible “Pavon” indicated the increased extent of AsA utilization to counter the higher amount of ROS produced under heat stress.

The central role of GSH in the antioxidant defense system is due to its ability to regenerate AsA through reduction of DHA in the AsA-GSH cycle [4,38] (Figure 9). Our results indicated a significant increase in the level of GSH in seedlings exposed to heat stress (Figure 4C) with a significantly higher accumulation in heat-tolerant “BG26” than others. This finding is in agreement with Tiwari and Yadav [109] and Kocsy et al. [110], a multi-fold increase in GSH content was recorded in tolerant maize and wheat varieties under heat stress exposure. They also suggested that an increase in GSH content under heat stress was due to a higher rate of GSH synthesis which was further accelerated by the enhanced GR activity. Similarly, in the present study, higher increase in the GSH content in “BG26” was well coordinated leading to an increased GR activity under heat stress (Figure 7A).

APX is a key enzyme in the AsA-GSH cycle and plays a vital role in plant defense against oxidative stress by catalyzing the conversion of H_2_O_2_ to water (Figure 9). Its essential role in the scavenging of H_2_O_2_ in chloroplasts, where CAT is absent, has been well established [111]. Our results showed a higher relative increase in APX activity during heat stress in the tolerant “BG26” and lower in heat susceptible “Pavon”, which is in agreement with Dash and Mohanty [112], Almeselmani et al. [77] and Tiwari and Yadav [109]. In addition, a significantly higher GR activity observed (Figure 7A) would complement APX in H_2_O_2_ scavenging [113].

Our results demonstrated that the activities of MDHAR and DHAR decreased markedly due to heat stress, which are partially supported by Rivero et al. [114]. The slight increase in APX and GR activities were not sufficient to protect the seedlings from ROS induced damages in susceptible variety “Pavon”. As the MDHAR and DHAR are equally important in regulating the level of AsA and its redox state under oxidative stress [93,115,116], decreases in the activities of these enzymes were followed by a decrease in AsA content; and these decreases was more pronounced in heat susceptible variety “Pavon” (Figure 4D). Interestingly, our results indicate that AsA–GSH cycle to be more efficiently operating in tolerant variety “BG26” than that of “Pavon” (Figure 9). The differential response of these varieties to heat stress as a consequence of variation in the levels of enzymatic and non-enzymatic antioxidants suggests that manipulation of AsA–GSH pathway may be a promising route to enhance heat tolerance in wheat.

## 5. Conclusions

In conclusion, a comprehensive analysis of the oxidative species and antioxidant interactions allowed us to capture the pathways that induced greater heat tolerance in “BG26” seedlings exposed to heat stress, compared to other varieties. The “BG26” was able to maintain highest dry matter and pigment stability, due to increased proline and glycine betaine levels, lower accumulation of H_2_O_2_, MDA and MG content and reduced oxidative stress with enhanced antioxidant capacity. Our findings demonstrated that heat-tolerant attributes are closely associated with the overall antioxidant activities to aid plants to maintain cellular homeostasis under heat stress. Our results establish a reference for further molecular analysis in wheat at the seedling stage, shaping the antioxidant system associated biochemical responses aimed to better adapt wheat to early-stage heat stress. Further investigations are needed to establish a pipeline for translating findings from seedling stage to different developmental stages including yield and yield-related parameters.

## Figures and Tables

**Table 1:** Leaf pigment contents of wheat varieties grown under control and heat stress conditions.

Variety	Growing Condition	Chl a (mg g^−1^ FW)	Chl b (mg g^−1^ FW)	Total Chl (mg g^−1^ FW)	Chl a/b Ratio	Carotenoid (mg g^−1^ FW) ^ǂ^	Anthocyanin (µg g^−1^ FW)^¶^
BG23	Control	0.49 ± 0.01 ^a,b^	0.26 ± 0.01 ^c,e^	0.75 ± 0.01 ^a–c^	1.88 ± 0.13 ^a^	0.09 ± 0.008 ^b–d^	71.51 ± 0.55 ^b^
	Heat stress	0.42 ± 0.01 ^c^	0.23 ± 0.01 ^e^	0.65 ± 0.01 ^d,e^	1.83 ± 0.12 ^a,b^	0.07 ± 0.012 ^c–e^	55.73 ± 0.47 ^d^
		(85.6 ± 1.45)	(88.8 ± 1.58)	(86.7 ±1.24)		(77.8 ± 5.88)	(78.0 ± 1.25)
BG25	Control	0.50 ± 0.02 ^a^	0.32 ± 0.02 ^a,b^	0.83 ± 0.02 ^a^	1.57 ± 0.12 ^a–c^	0.13 ± 0.015 ^a,b^	80.40 ± 0.67 ^a^
	Heat stress	0.42 ± 0.01 ^c^	0.28 ± 0.01 ^b–e^	0.70 ± 0.02 ^c,d^	1.50 ± 0.01 ^b,c^	0.11 ± 0.012 ^b,c^	67.40 ± 0.50 ^c^
		(83.5 ± 0.83)	(87.2 ± 5.61)	(84.7 ± 1.67)		(84.6 ± 1.04)	(83.9 ± 1.26)
BG26	Control	0.44 ± 0.01 ^b,c^	0.35 ± 0.01 ^a^	0.79 ± 0.01 ^a,b^	1.26 ± 0.03 ^c^	0.17 ± 0.009 ^a^	79.25 ± 0.83 ^a^
	Heat stress	0.38 ± 0.01 ^c,d^	0.31 ± 0.01 ^a–c^	0.70 ± 0.01 ^c,d^	1.23 ± 0.08 ^c^	0.15 ± 0.014 ^a,b^	67.56 ± 0.59 ^c^
		(87.2 ± 2.58)	(89.5 ± 2.61)	(88.2 ± 0.35)		(88.2 ± 8.48)	(85.3 ± 0.77)
Pavon	Control	0.43 ± 0.01 ^b,c^	0.30 ± 0.01 ^a–d^	0.73 ± 0.02 ^b,c^	1.42 ± 0.05 ^c^	0.05 ± 0.012 ^d,e^	71.21 ± 0.74 ^b^
	Heat stress	0.32 ± 0.02 ^d^	0.25 ± 0.01 ^d,e^	0.58 ± 0.02 ^e^	1.28 ± 0.10 ^c^	0.03 ± 0.009 ^e^	50.74 ± 0.65 ^e^
		(75.3 ± 4.41)	(83.6 ± 4.88)	(78.6 ± 1.62)		(60.0 ± 8.45)	(71.3 ± 0.91)

Values represent as mean ±SE. Figures in the parentheses indicate stability of that pigment. Values in a column with different letter(s) are significantly different at *p* ≤ 0.05. ^ǂ^ lutein equivalent, and cyanidin-3-glucoside equivalent.

## Data Availability

The data that support the findings of this study are available from the corresponding author upon reasonable request.

## Supplementary Materials

The following are available online at https://www.mdpi.com/2076-3921/10/3/351/s1, Figure S1: Comparative changes in shoot and root length of wheat varieties under control and heat stressed conditions; Table S1: Cross and pedigree of released wheat varieties in Bangladesh; Table S2: Stress tolerance index (STI) of studied variables; Table S3. Variance components (%) of morphological, physiological and biochemical variables in the context of wheat varieties × heat stress using the general linear model and Table S4. Morphological variables of wheat varieties grown under control and heat stress conditions. SL- shoot length, RL- root length, SDM- shoot dry matter, RDM- root dry matter, TDM- total dry matter.

## Abbreviations:

AGE	Advanced glycation end product
Anth	Anthocyanins
AO	Ascorbate oxidase
APX	Ascorbate peroxidase
AsA	Ascorbate
BARI	Bangladesh Agricultural Research Institute
BSA	Bovine serum albumin
Car	Carotenoids
CAT	Catalase
CDNB	1-chloro-2,4-dinitrobenzene
Chl	Chlorophyll
DHA	Dehydroascorbate
DHAR	Dehydroascorbate reductase
DTNB	5,5′-dithiobis-(2-nitrobenzoic acid)
EDTA	Ethylenediaminetetraacetic acid
GB	Glycine betaine
Gly I	Glyoxalase-I
Gly II	Glyoxalase-II
GPX	Glutathione peroxidase
GR	Glutathione reductase
GSH	Glutathione (reduced)
GSSG	Glutathione (oxidized)
GST	Glutathione s-transferase
IPCC	The Intergovernmental Panel on Climate Change
LOX	Lipoxygenase
LTD	Leaf temperature depression
MDA	Malondialdehyde
MDHA	Monodehydroascorbate
MDHAR	Monodehydroascorbate reductase
MG	Methylglyoxal
MII	Membrane injury index
NADPH	Nicotinamide adenine dinucleotide phosphate (reduced)
NBT	Nitroblue tetrazolium
POD	Guaiacol peroxidases
Pro	Proline
PUFA	Polyunsaturated fatty acid
RDM	Root dry matter
RH	Relative humidity
RL	Root length
ROS	Reactive oxygen species
SDM	Shoot dry matter
SL	Shoot length
SLG	S-D-lactoylglutathione
SOD	Superoxide dismutase
STI	Stress tolerance index
TBA	Thiobarbituric acid
TCA	Trichloroacetic acid
TDM	Total dry matter
