# Supplementary material for: Physiological and Biochemical Dissection Reveals a Trade-Off between Antioxidant Capacity and Heat Tolerance in Bread Wheat (Triticum aestivum L.)
Source: Antioxidants (Basel). 2021 Feb 26;10(3):351. doi: 10.3390/antiox10030351 (PMC7996931; doi:10.3390/antiox10030351)
Supplement: Supplementary file 1 [file antioxidants-10-00351-s001.pdf]

Table S1. Cross and pedigree of released wheat varieties in Bangladesh

Variety	Pedigree	Year of released	Yield (t ha^{-1})
Pavon 76	VCM//CNO/7C/3/KAL/BB<br>CM 8399-D-4M-3Y-1M-IY-IM-0Y-0BGD	1979	3.70
BARI Gom 23	NL297*2/LR25	2005	4.65
BARI Gom 25	ZSH 12/HLB 19//2*NL297	2010	4.30
BARI Gom 26	ICTAL 123/3/RAWAL 87//VEE/HD 2285<br>BD(JO)9585-0JO-3JE-0JE-0JE-HRDI-RC5DI	2010	4.30

Table S2. Stress tolerance index (STI) of studied traits.

Variety	Stress tolerance index (STI)									
	**Chl a**	**Chl b**	**T Chl**	**Chl a/b**	**Car**	**Anth**	**LTD**	**MII**	**MG**	**H_2O_2**
BG 23	0.95	0.63	0.81	1.46	0.52	0.70	0.82	1.45	2.00	1.44
BG 25	0.97	0.95	0.97	1.00	1.18	0.95	1.52	1.16	1.32	1.16
BG 26	0.77	1.15	0.92	0.65	2.11	0.94	1.92	0.65	1.17	1.27
Pavon	0.64	0.79	0.70	0.78	0.12	0.63	0.09	1.78	2.28	2.30

Variety	Stress tolerance index (STI)									
	MDA	LOX	Pro	GB	GSH	AsA	Gly-I	Gly II	SOD	CAT
BG 23	1.55	1.23	1.12	2.11	1.53	0.61	0.97	1.05	1.35	0.67
BG 25	1.15	1.63	1.37	3.10	1.49	0.69	1.43	1.50	1.20	0.90
BG 26	1.06	1.41	1.40	2.98	2.18	0.97	1.55	1.71	1.40	1.12
Pavon	2.83	1.91	1.04	0.89	1.17	0.35	0.88	0.51	0.88	0.49

Variety	Stress tolerance index (STI)							
	POD	GPX	GST	GR	APX	DHAR	MDHAR	TDM
BG 23	0.84	1.64	1.25	1.64	1.32	0.83	0.78	0.70
BG 25	0.74	1.39	1.20	1.75	1.48	0.82	0.65	1.13
BG 26	1.16	2.10	1.60	2.36	1.90	1.06	1.15	0.93
Pavon	0.66	0.94	0.81	1.45	1.07	0.71	0.57	0.66

Table S3. Variance components (%) of morphological, physiological and biochemical traits in the context of wheat varieties × heat stress using the general linear model

Trait	Variety (V)	Treatment (T)	V × T	Residual
SL	34.4***	59.9***	4.7*	1.0***
RL	13.9**	83.3***	1.2	1.6***
SDM	28.4***	64.1***	6.4**	1.2***
RDM	47.7***	46.7***	5.0**	0.7***
TDM	33.8***	60.0***	5.6**	0.7***
Chl *a*	20.2***	76.9***	1.6	1.2***
Chl *b*	44.2***	52.4***	0.8	2.5***
T Chl	13.8***	84.3***	1.2	0.6***
Chl *a*/*b*	87.8***	6.1	0.6	5.4***
Car	82.0***	15.9*	0.1	2.1***
Anth	15.8***	82.8***	1.3***	0.1***
LTD	93.8***	3.1	0.1	3.0***
MII	48.3***	45.4***	6.0***	0.2***
MG	9.8***	87.8***	1.7	0.7***
H2O2	14.9***	78.7***	6.4***	0.1***
MDA	22.8***	67.7***	9.4***	0.1***
LOX	6.4***	84.1***	9.2***	0.3***
Gly I	39.1***	57.2***	2.8*	0.8***
Gly II	72.0***	20.7*	1.4	6.0**
Pro	11.4***	82.6***	5.8***	0.3***
GB	12.1***	82.5***	5.1***	0.3***
GSH	9.8***	83.3***	6.9***	0.1***
AsA	12.5***	82.1***	5.2***	0.2***
SOD	23.1***	70.0***	6.5***	0.5***
CAT	30.6***	65.5***	2.1	1.8***
POD	33.5***	62.7***	1.4	2.4***
GPX	15.6*	76.2**	2.1	6.1*
GST	36.8***	61.0***	2.1***	0.1***
APX	10.8*	80.8***	4.7	3.7***
GR	4.3***	93.6***	2.0***	0.1***
MDHAR	25.1***	71.3***	2.0	1.6***
DHAR	18.4***	75.3***	6.0***	0.4***

SL- shoot length, RL- root length, SDM- shoot dry matter, RDM- root dry matter, TDM- total dry matter, Chl *a*- chlorophyll *a*, Chl *b*- chlorophyll *b*, T.Chl- total chlorophyll, Chl *a*/*b*- chlorophyll a/b, Car- carotenoids, Anth- anthocyanin, LTD- leaf temperature depression, MII- membrane injury index, MG- methylglyoxal, MDA- malondialdehyde, H_2O_2- hydrogen peroxide, LOX- lipoxygenase, Pro- proline, GB- glycine betaine, GSH- glutathione, AsA- ascorbate, Gly I- glyoxalase I, Gly II- glyoxalase II, SOD- superoxide dismutase, CAT- catalase, POD- peroxidase, GPX- Glutathione peroxidase, GST- Glutathione *S*-transferases, GR- glutathione reductase, APX- ascorbate peroxidase, MDHAR- monodehydroascorbate reductase, and DHAR- dehydroascorbate reductase.

***, ** and * indicate significant at P≤0.001, P≤0.01 and P≤0.05, respectively.

Table S4. Morphological traits of wheat varieties grown under control and heat stress conditions. SL- shoot length, RL- root length, SDM- shoot dry matter, RDM- root dry matter, TDM- total dry matter

Variety	Treatment	SL (cm)	RL (cm)	SDM (mg)	RDM (mg)	TDM (mg)
BG23	Control	12.8±0.73[cd]	17.9±0.82[a-c]	16.1±0.55[a-c]	11.2±0.28[c]	27.4±0.81[b]
	Heat stress	10.9±0.62[d]	14.4±0.65[d]	13.7±0.59[c]	8.5±0.33[d]	22.2±0.91[c]
		(15.4)	(19.7)	(15.1)	(24.6)	(19.0)
BG25	Control	18.1±0.35[a]	21.1±0.46[a]	18.4±0.55[a]	14.6±0.09[a]	32.9±0.50[a]
	Heat stress	15.8±0.52[ab]	17.9±0.87[a-c]	16.5±0.60[ab]	13.4±0.31[ab]	29.9±0.61[ab]
		(12.8)	(15.3)	(10.2)	(8.0)	(9.21)
BG26	Control	14.7±0.10[bc]	18.4±0.51[ab]	16.1±0.47[a-c]	13.3±0.32[ab]	29.3±0.62[b]
	Heat stress	12.9±0.43[cd]	15.8±0.52[b-d]	14.8±0.49[bc]	12.8±0.23[bc]	27.6±0.48[b]
		(12.0)	(14.1)	(7.9)	(3.8)	(6.0)
Pavon	Control	16.3±0.71[ab]	19.5±0.88[a]	15.7±0.41[bc]	12.5±0.58[bc]	28.2±0.96[b]
	Heat stress	11.3±0.29[d]	14.9±0.58[cd]	10.6±0.35[d]	9.6±0.23[d]	20.2±0.20[c]
		(31.1)	(23.5)	(32.3)	(23.7)	(28.5)

Values represented as mean ±SE. Values in the parentheses indicate percent decrease over normal. Values in a column with different letter(s) are significantly different at P≤0.05.

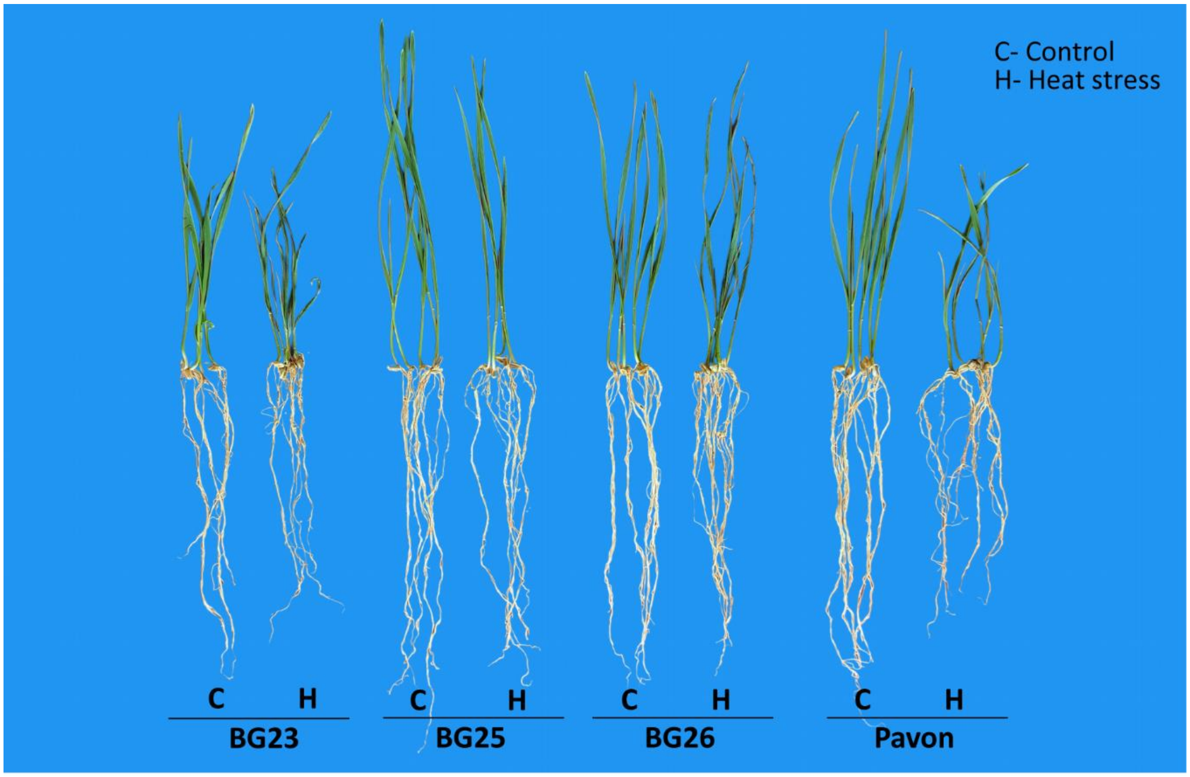

Fig. S1. Comparative changes in shoot and root length of wheat varieties under control and heat stressed conditions.
